# Three Dimensional Visualization and Fractal Analysis of Mosaic Patches in Rat Chimeras: Cell Assortment in Liver, Adrenal Cortex and Cornea

**DOI:** 10.1371/journal.pone.0031609

**Published:** 2012-02-07

**Authors:** Stephen Iannaccone, Yue Zhou, David Walterhouse, Greg Taborn, Gabriel Landini, Philip Iannaccone

**Affiliations:** 1 Children's Memorial Research Center, Northwestern University, Chicago, Illinois, United States of America; 2 Center for Reproductive Medicine and Fertility, The University of Chicago, Chicago, Illinois, United States of America; 3 School of Dentistry, University of Birmingham, Birmingham, United Kingdom; Brigham and Women's Hospital, United States of America

## Abstract

The production of organ parenchyma in a rapid and reproducible manner is critical to normal development. In chimeras produced by the combination of genetically distinguishable tissues, mosaic patterns of cells derived from the combined genotypes can be visualized. These patterns comprise patches of contiguously similar genotypes and are different in different organs but similar in a given organ from individual to individual. Thus, the processes that produce the patterns are regulated and conserved. We have previously established that mosaic patches in multiple tissues are fractal, consistent with an iterative, recursive growth model with simple stereotypical division rules. Fractal dimensions of various tissues are consistent with algorithmic models in which changing a single variable (e.g. daughter cell placement after division) switches the mosaic pattern from islands to stripes of cells. Here we show that the spiral pattern previously observed in mouse cornea can also be visualized in rat chimeras. While it is generally held that the pattern is induced by stem cell division dynamics, there is an unexplained discrepancy in the speed of cellular migration and the emergence of the pattern. We demonstrate in chimeric rat corneas both island and striped patterns exist depending on the age of the animal. The patches that comprise the pattern are fractal, and the fractal dimension changes with the age of the animal and indicates the constraint in patch complexity as the spiral pattern emerges. The spiral patterns are consistent with a loxodrome. Such data are likely to be relevant to growth and cell division in organ systems and will help in understanding how organ parenchyma are generated and maintained from multipotent stem cell populations located in specific topographical locations within the organ. Ultimately, understanding algorithmic growth is likely to be essential in achieving organ regeneration in vivo or in vitro from stem cell populations.

## Introduction

The development of mammalian organs requires key steps. First, the parenchyma mass must form from appropriate cells in the right location and at the right time. Once the mass is formed, the cells must divide and expand to produce the parenchyma compartment. With carefully regulated expansion, growth and differentiation the primordial tissue develops into a functional organ [1].

Examining mosaic patches (aggregates of cells of the same parental lineage in tetraparental mosaic animals known as chimeras) in some tissues reveals that the cellular patterns formed are complex in geometry and have characteristic fractal dimensions [2]–[6]. For example, we showed that characteristic fractal dimensions are associated with liver growth and modeled growth as a procession of regular and iterative rule based cell division [4]. Stereotypical and iterative rules for cell division may be the way rapid organ growth could be regulated by just a few genes. Examining mosaic pattern was also used to establish that preneoplastic lesions and cancer in the liver are clonal, thus develop from single cells as a result of stochastic processes [7]–[9].

The forces that create the mosaic pattern; cell division, cell movement and cell death can be simulated and then compared with mosaic pattern observed in animals [10]. In the liver, cell divisions can be modeled by random placement of daughter cells, which is sufficient to create realistic patterns. In simulations of growth of the adrenal cortex the placement of daughter cells must be biased by a force (e.g., cell adhesion) during division that tends to keep daughters cells spatially contiguous in order to create a realistic pattern. Importantly, fractal dimension is a useful tool to express the differences between liver and adrenal growth patterns [10]. Previous work with rat chimeras combined embryonic tissues from genetically distinguishable congenic strains differing at the RT-1 locus. Monoclonal antibodies that were radioactively labeled were used to determine the parental lineage (strain of origin) of specific cells and tissues. Fluorescent proteins such as enhanced green fluorescent protein (eGFP) provide the opportunity to examine mosaic patches with much more clarity and resolution utilizing confocal microscopy than with previous techniques. Here we show that the liver and adrenal glands from rat chimeras with eGFP markers show the same underlying distribution of cells in patches as we have shown previously with other markers but that the data can be obtained with single cell resolution.

The cornea presents a spiral pattern previously shown in mouse [11] that we show here is conserved in the rat. The cornea grows from limbal stem cells (LSC) and a commonly held view is that the progeny of the LSC either migrate centripetally or are left behind as a constrained trail of cell division history thus creating the pinwheel pattern. However, a number of fine detail aspects of the patterns observed here in the rat and previously in the mouse, including a central whorl and branching are not explained by stem cell mechanics alone. Moreover, a patchy island-like pattern persists for about two months in both the mouse and the rat and its fairly rapid transition to a spiral pattern is not easily explained by stem cell division pattern driven mechanisms.

Here we show that patterns observed in the mouse cornea are also seen in the rat in unbiased (i.e., the development of the tissue and mosaicism are independent) aggregation chimeras using markers that are independent of the corneal epithelium. This supports the contention that the patterns are representative of fundamental processes of consequence to correct tissue construction. We show that the transition between fundamentally distinct patterns in the rat cornea that occurs rapidly at about 2–3 months of age can be detected by plotting fractal dimension over time. We demonstrate computationally that spiral patch patterns are most like loxodromes, pathways to the pole of a sphere with invariant heading.

## Materials and Methods

### Chimeras

To generate animals with unequivocal genetic markers we produced chimeric rats by morula amalgamation [1], [12]–[14]. SD and SD-eGFP strains of rat were used such that two morulae, one from each strain, were aggregated with established procedures. The SD-eGFP strain is a transgenic produced by injection of lentivirus (FUGW, the kind gift of Carlos Lois, MIT) into the perivitelline space of one-cell embryos with subsequent transfer to pseudopregnant surrogate mothers. FUGW utilizes the human polyubiquitin-C promoter which has been shown to drive robust expression of transgenes [15]. The SD-eGFP strain used was selected because of its ubiquitous, uniform, stable high expression of eGFP in tissues. Liver sections from seven transgenic animals were examined with confocal microscopy (described below) and in 14 sections all but two were uniformly (100%) fluorescent. The adrenal glands were examined in three of the animals; these showed evidence of transgene silencing (up to 30% of the total area was non-fluorescent in one animal) as has been previously reported [16]. Corneas were examined in three transgenic animals and one of them showed a coherent area, less than 1% of the total area lacking fluorescence. The others were uniformly fluorescent. Some minor variations in signal intensity were noted. In preliminary experiments we combined eight pairs of morulae where each partner was transgenic. Four of these pairs developed into blastocysts and all were completely fluorescent from GFP expression as predicted.

**Figure 1:**
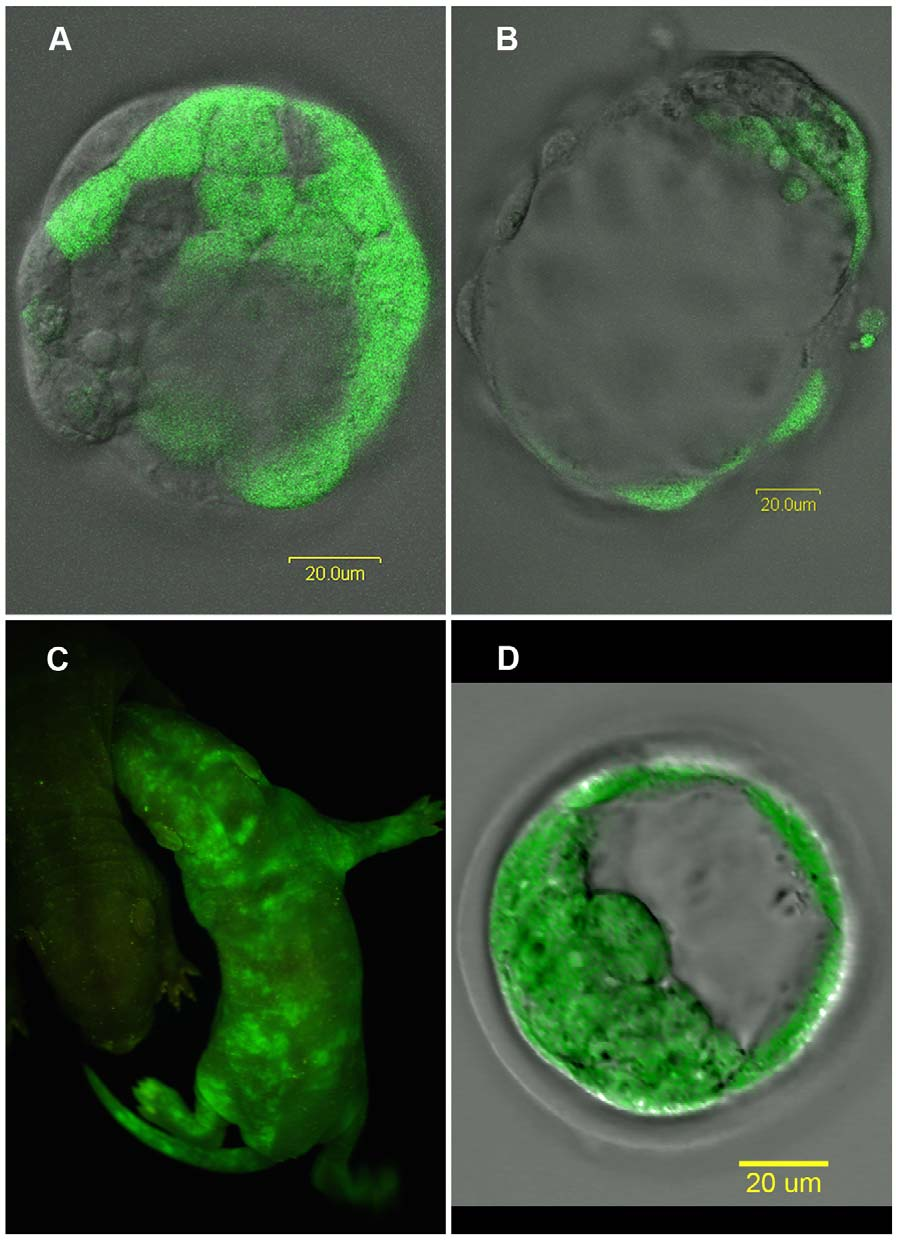
Production of rat chimeras. Rat chimeras were produced by morulae aggregation where one morula is from a transgenic eGFP lineage and the other is of wild type lineage. (A) These distinct cell lineages can be seen in the embryos before implantation. (B) Further culturing of these embryos reveals the labeled cells integrating into the inner cell mass. (C) A newborn chimera pup, right half of photograph, can be easily distinguished from a non-chimeric littermate, barely seen in the left half of the photograph, when visualized under blue light. (D) A transgenic eGFP blastocyst. Some areas of the transmitted image, where the light isn't restricted by the confocal aperture around the zona pelucida and the periphery of the blastocyst are out of focus which doesn't occur in the fluorescent image where all cells in the inner cell mass as well as the trophectoderm are clearly labeled with eGFP. Scale bars in (A), (B) and (D) are 20 µm.

Thus, one of the morulae of each of the pairs was modified genetically such that it produced enhanced green fluorescent protein (eGFP). When this was paired with a wild type morula, they combined to form a chimeric rat embryo (Figure 1). Use of a fluorescent microscope allowed for the visualization of the marked cells. All animal work was conducted in our Association for Assessment and Accreditation of Laboratory Animal Care International (AAALAC) accredited facilities with Institutional Animal Care and Use Committee (IACUC) approval (protocol 95018).

### Visualization

The rat chimeras were identified and photographed using intense blue LED illumination (BLS Ltd, Budapest, Hungary). Tissues obtained from the chimeras were examined and imaged with a Zeiss META 510 confocal laser-scanning microscope. The data were imported and rendered with Volocity software (Perkin Elmer, Inc., U.S.A.).

### Tissues

The tissues were prepared by first thoroughly perfusing the animal to remove red blood cells from the tissue, which otherwise could cause autofluorescence related signal interference. Next, the animal was fixed using 4% paraformaldehyde by perfusion. The organs were then dissected and stored in 4% paraformaldehyde overnight and then in 1% paraformaldehyde.

One day prior to sectioning, the tissues were transferred to a 30% sucrose solution. The tissue was mounted and frozen then cut at 35 µm on a Leica (CM 3050 S) cryostat. Adjacent serial sections were prepared and mounted on glass slides with PBS then stored in a humidity chamber to prevent them from drying out. For preparation of the corneas the eyes were stored in 4% paraformaldehyde and the corneas dissected, flattened with short radial cuts and mounted with PBS under a glass slide in an optical quality glass-bottom culture dish (World Precision Instruments).

### Microscopic Imaging

Slides were imaged through a 25 X Zeiss multi-immersion 0.8 NA objective on a Zeiss 510 inverted fluorescent confocal microscope equipped with a motorized stage. Using the motorized stage we could capture many fields of view and tile them together to form a large montage of the section. This allowed us to capture areas larger than 4 mm by 4 mm without sacrificing the resolution necessary for analysis.

The images were histogram stretched to yield the best dynamic range for the signal. A median filter was applied to remove any potential noise from the acquisition of the image. Once the image was binary (all pixels were either black or white, or thought of as “off” or “on”, respectively), the image was analyzed to determine the fractal dimension as described below.

The images collected from the liver and adrenal gland were processed further to prepare them for three-dimensional reconstruction. The images were aligned in serial and then loaded into software to form a 3D model of the sections (Volocity, Perkin Elmer).

Surface and mass fractal dimension were established with a variety of methods [4], [10]. For example, surface fractal dimension of the adrenal cortical patches was previously determined by using the yardstick method [17] where yardsticks of different lengths (e) were used to measure the length of the perimeter of a patch (L(e)). Then fractal dimension (D) was determined as 1 - the slope of the regression of log L(e) plotted against log (e). Here we used the box counting method [18] where a grid of boxes of various sizes is applied to the object and the number of boxes required to cover the perimeter of the object is determined for a large number of box sizes. The slope of the log-log plot of box size versus the number of boxes occupied by the object's perimeter is –D. We have shown previously that there is excellent agreement between surface fractal dimensions determined by box counting and the yardstick method.

### Hemisphere projection

The coordinate (x,y) data points of patch edges were collected from the original cornea images and entered into MS Excel. These data were used to calculate the projected cylindrical coordinates in 3D, ρ is the distance from the longitudinal axis, φ is the angular coordinate, and z is the height along the longitudinal axis.

**Figure 2:**
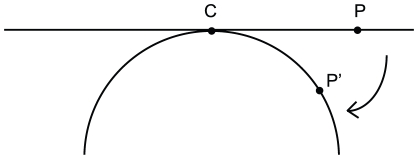
Projection of rat chimera corneal image onto a hemisphere. The cornea image, seen as the line across the top of the figure, was centered at the apex of a hemisphere, seen as the arc beneath the line, at point C. The location of the projected point P' on the hemisphere was calculated from the distance of the original point P on the image such that arclength CP' is equal to the original length CP.

To project the image onto the hemisphere, a point's distance from the center of the image was set to the arclength from the apex of the hemisphere (Figure 2).

Its angular component remained consistent, and its projected height was calculated from the arclength, which corresponds to how far down the point was draped. The following equations were used for the calculations:
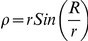


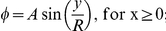


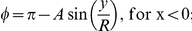


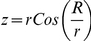
Where 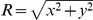, the distance of the point from the center of the image, and r is the radius of the hemisphere.

These projected data points were put into Apple's Grapher program to make a 3D model of the corneal patches.

### Spiral Curve Fitting

Fitting curves to the patch edges seen in the chimeric corneas consisted of several steps. First starting with general equations we described the geometry of the patches. Chaudhuri, et al. [19] reported that while the conic constant, a measure of the eccentricity of the surface, of an individual rat's anterior cornea is closer to an ellipse, the mean conic constant of many individuals is closer to zero and therefore may be better fit by circles. To generalize the corneas of all animals examined, we started with the assumed surface of a sphere and patch edges as spherical spirals defined by the following parametric equations:
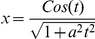


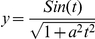


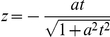



The flattened corneas produced two-dimensional images while the curve traced by a spherical spiral is three-dimensional, so to allow comparisons the idealized curves were transformed. A stereographic projection of the spherical spiral produces a two-dimensional curve suitable for fitting to the cornea images and is a logarithmic spiral. These transformed spirals were used as the basis for curve fitting the patch edges, and the stereographic projection was performed as follows:
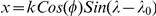


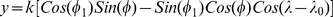
Where,
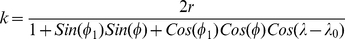
and λ is the inclination, λ_0_ is the central inclination, φ is the azimuth, and φ_1_ is the central azimuth (set to π/2) of projection as described in the Wolfram MathWorld page on Stereographic Projection [20] (Weisstein, Eric W. “Stereographic Projection.” From *MathWorld*–A Wolfram Web Resource. http://mathworld.wolfram.com/StereographicProjection.html).

Since this projection requires the data points to be in spherical coordinates (radius, inclination, and azimuth) instead of the Cartesian coordinates yielded from our equations of the spherical spiral equations, they were transformed as follows:
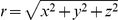


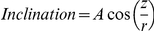


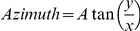



The fitting of the spiral to the data was achieved visually by adjusting the parameters of the equations, *a* and *λ_0_*. An affine shift was introduced to account for error in the assumed position of the center of the cornea's spiral as well as an independent stretching factor in x and y to account for an observed eccentricity. This eccentricity could be due to non-uniform eye shape in the animal discussed above and in Chaudhuri, et al. [19] or due to directional bias of cellular or patch movement.

Finally, selected points were manually transcribed and overlaid onto the original data using Photoshop to show the relation between the fitted spirals and the original cornea patch data.

Some patch edges were compared to logarithmic spirals by measuring the distance from the assumed center of the spiral to the patch edge at arbitrary points. For a logarithmic spiral the distance should increase exponentially as one moves along the curve. These measurements were compared to those obtained in the same way from an ideal logarithmic spiral and to an Archimedean spiral. The results were plotted as distance (vector length) vs. the angle (theta) between the line from the center of the spiral to the innermost point along the edge of the spiral and lines from the center of the spiral to arbitrary points further along the spiral. The correlation coefficients (r^2^) of the fit to an exponential curve were calculated and compared to r^2^ of the linear fits. One turn of the spiral is 2 pi radians or 360 degrees. The calculations and plots were done using Excel.

## Results

### Chimeras

Figure 1 shows the production of chimeras. The mixture of cells in the developing embryo is evident by the blastocyst stage. The inner cell masses also show mixtures of eGFP marked and unmarked cells. This is also reflected in the newborn animals shown in the figure. Patchy expression of eGFP (green) in the skin is evidence of chimerism.

### Liver and Adrenal

**Figure 3:**
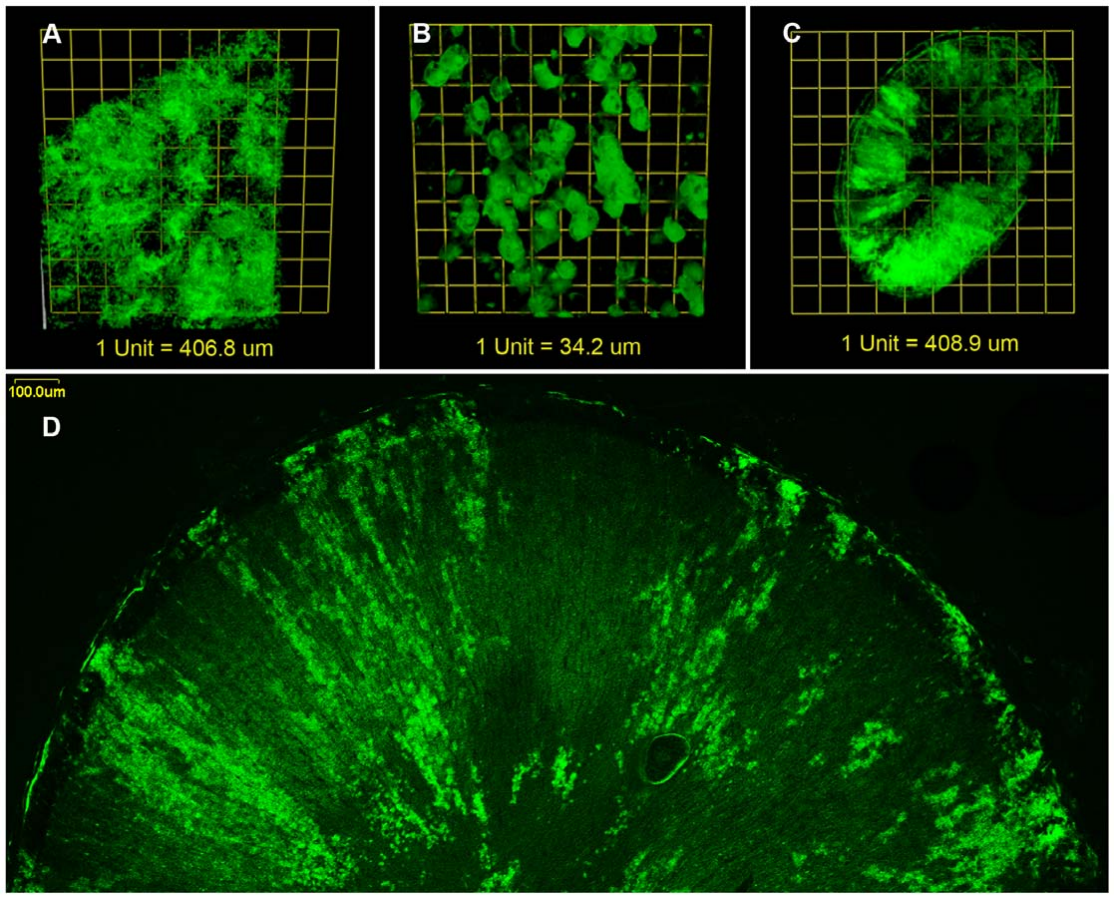
Three-dimensional reconstruction of chimeric rat liver and adrenal gland. (A) Liver from a chimeric rat was sectioned at 35 µm and imaged with confocal microscopy. One plane of focus was used to represent each 35 µm section. These sections were then stacked and aligned to render a three dimensional model illustrating the complex interconnected nature of the patches. The total area shown is approximately 4 mm by 4 mm with the eGFP lineage shown as green. (B) A higher magnification view of liver from a chimeric rat, the three-dimensional rendering was produced in a similar fashion as in (A) except that it was imaged under higher power to illustrate the detail of fluorescent patches with a total area shown approximately 0.35 mm by 0.35 mm. (C) This process of rendering a three dimensional model was repeated with cross sections of the adrenal gland. This highlights the radial cord-like structure of the fluorescent patches in the adrenal cortex, which are reminiscent of pencils in a cup. The total area shown is approximately 4 mm by 4 mm. (D) Cross section through the chimeric adrenal gland illustrates two types of patches: the clonally derived radial cord-like patterning of the interior of the cortex, and the stem-like appearance of the outer surface. The scale bar is 100 µm. Shown in (A), (B), and (C) are the first frames of animations of the rendered models being rotated. The full movies can be seen in Figure S1, Figure S2, and Figure S3 respectively.

Previous studies have shown that the mixtures of cells in the adult organs are variable in proportion from tissue to tissue but are arrayed in some tissues in characteristic patterns. This result is obtained with SD<->SDeGFP rat chimeras as well. Figure 3A shows the characteristic islands in a sea appearance of the liver.

The fractal nature of the patches of lineage related cells is well established. The implication of the extension of these patterns into 3 dimensions suggests that highly complex interconnected patches would be obtained at some critical proportion of marked cells. Previous studies with computer simulations indicate that this is likely to occur at proportions of marked cells close to 50% [21]. Figure 3A and Figure 3B show examples of 3 dimensional reconstructions of liver sections from rat chimeras. For a more detailed look, Figures S1 and S2 show the reconstructions of the liver being rotated in 3 dimensions.

The adrenal cortex of chimeras comprises cords of cells that originate from the outside of the organ and extend inwardly during development (Figure 3C, D). There are several possible ways in which this growth could occur. Three dimensional reconstruction of the organ (Figure 3C) allows us to conclude that the cords are arrayed like pencils in a cup from random patches oriented on the outside of the organ and inward growth with biased daughter cell positioning [22], [23]. This strongly implies that growth is algorithmic [23]. The eGFP marked chimeras replicate previous results with different markers in the liver and the adrenal gland. Figure S3 is an animation of the reconstruction of the adrenal gland in 3 dimensions.

### Cornea

**Figure 4:**
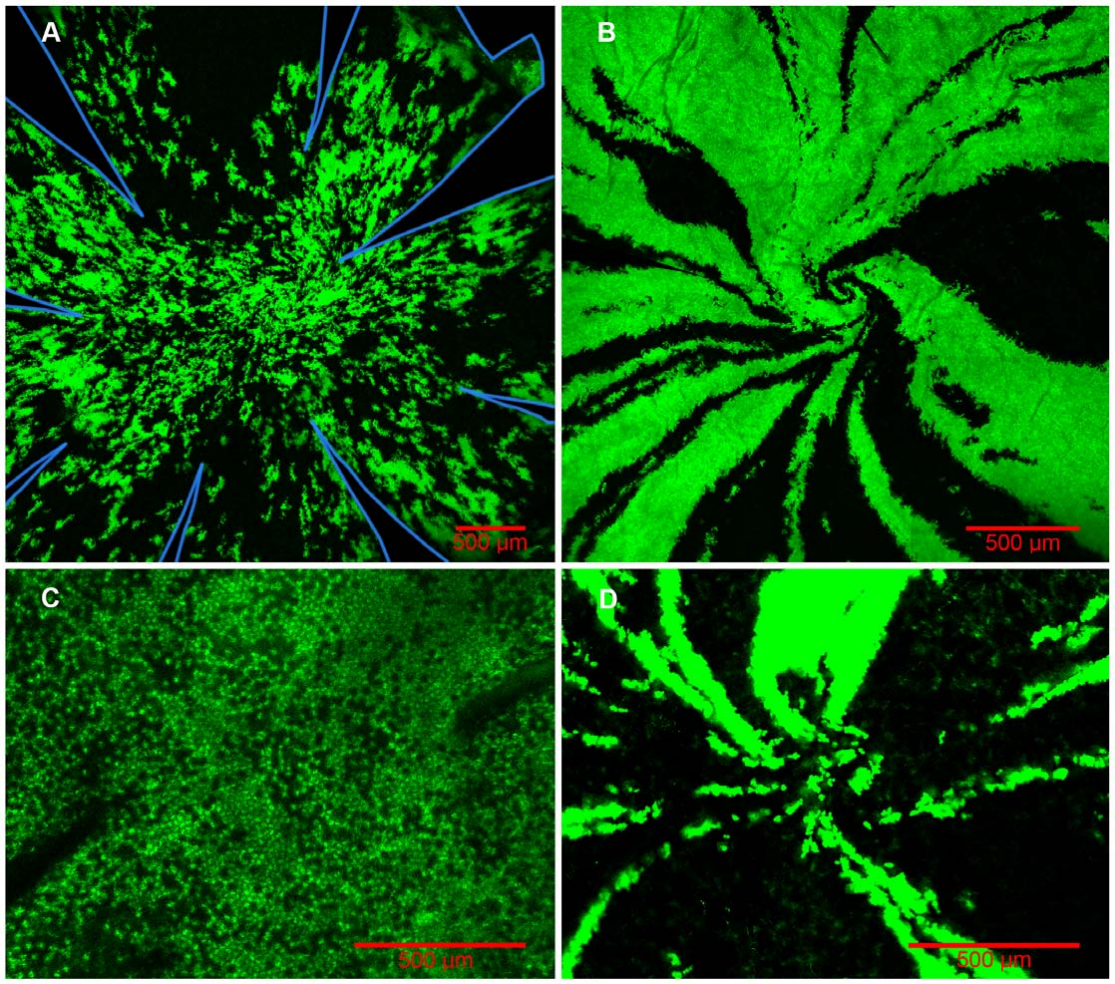
Chimeric rat corneas. (A) In young rat chimeras corneas display a geographic pattern reminiscent of islands in the sea, which is indicative of unconstrained growth of a stem cell like nature shown here from a 3 week-old animal. Areas where the cornea has been cut to relax it are outlined in blue. (B) This pattern transitions into a highly constrained pinwheel pattern in the adult rat illustrated here in a 16 month-old animal. (C) This pinwheel pattern is not present in the endothelial layer of the cornea. (D) Endothelial pattern is unrelated to that of the epithelium of the same cornea. The epithelium shown in (D) also displays several alternate patterns including arcs and branches in the pattern. eGFP lineage is green. Scale bars = 500 µm.

Another example of constrained growth in the organs of these animals is in the cornea. As the cornea develops there are two distinct growth patterns. One is like the liver, islands in a sea, while the other is like the adrenal cortex with stripes of cells coherently of the same lineage. The pattern is further modified by a pinwheel effect (Figure 4). This pattern is highly reminiscent of spiral phylotaxis, for example, the systematic array of seeds or florets in a flower [24].

**Figure 5:**
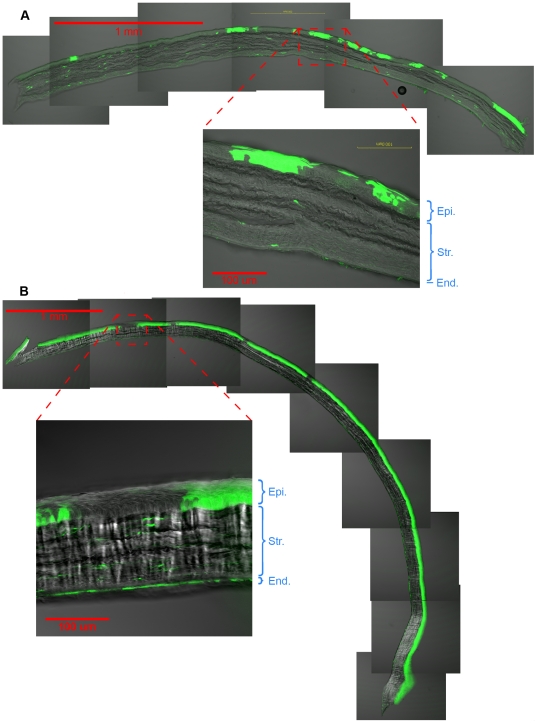
Cross sections of chimeric rat corneas. (A) Cross sections of chimeric rat corneas reveal that there is no concordance between patches of cells labeled in the epithelium and those labeled in the endothelium. (B) Also apparent is the lack of extensive lateral mixing of cells in the epithelial layer. Cells form mostly contiguous patches through the thickness of the epithelium. Epi. is the epithelium, Str. is the stroma, and End. is the endothelium. eGFP lineage is green.

The spiral pattern comprises epithelial patches with little lateral mixing across the boundary (Figure 5). The endothelium in contrast does not show a constrained spiral pattern but one more like the liver (Figure 4C). In addition, on antero-posterior sections through the corneal thickness, the patches in the endothelium also appear unrelated to those of the epithelium (Figure 5).

### Fractal Dimension

Thirty corneas were examined from a total of 20 rat chimeras varying in age from eight days to 18 months of age. Fourteen corneas from nine animals displayed a well-developed spiral pattern; six corneas had a clockwise spiral and eight corneas a counter-clockwise spiral. Of the nine animals with spiral patterns both corneas were examined in five and of these four animals had opposite handedness in the spiral patterns in their corneas. In two of the 20 rat chimeras the three corneas examined had a striped pattern with little or no indication of spiral curvature. Two corneas from one animal could not be evaluated. The remaining 11 corneas from eight animals (all under 2 months of age) were patchy.

**Figure 6:**
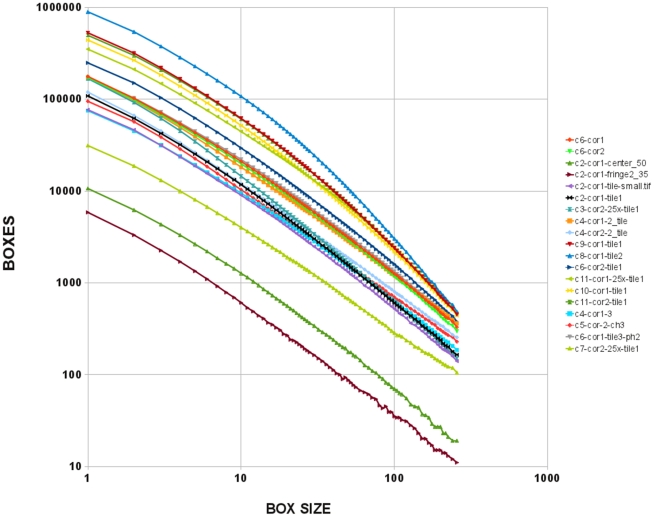
Fractal dimension of chimeric rat corneas. Once images of the chimeric rat corneas are turned into binary images, the fractal dimension is determined from the slope of the regression line of box counting data. The box counting data is collected over several orders of magnitude in size and regression lines are fitted to log-log plots of the number of boxes counted versus the size of the box. Shown here are the log-log plots of 19 images from 10 chimeric rats, demonstrating that they are fractal.

The patches are fractal and the surface fractal dimension can be calculated (Figure 6). Overall the average surface fractal dimension from 34 samples of 30 individual corneas obtained from 20 chimeras was 1.31. Control corneas from two transgenic and two non-transgenic rats show predicted expression of eGFP. However, in less than 1% of the area of GFP transgenic corneas unexpected coherent areas without eGFP expression was observed. In some of the transgenic corneas minor variation in expression levels of GFP were observed.

**Figure 7:**
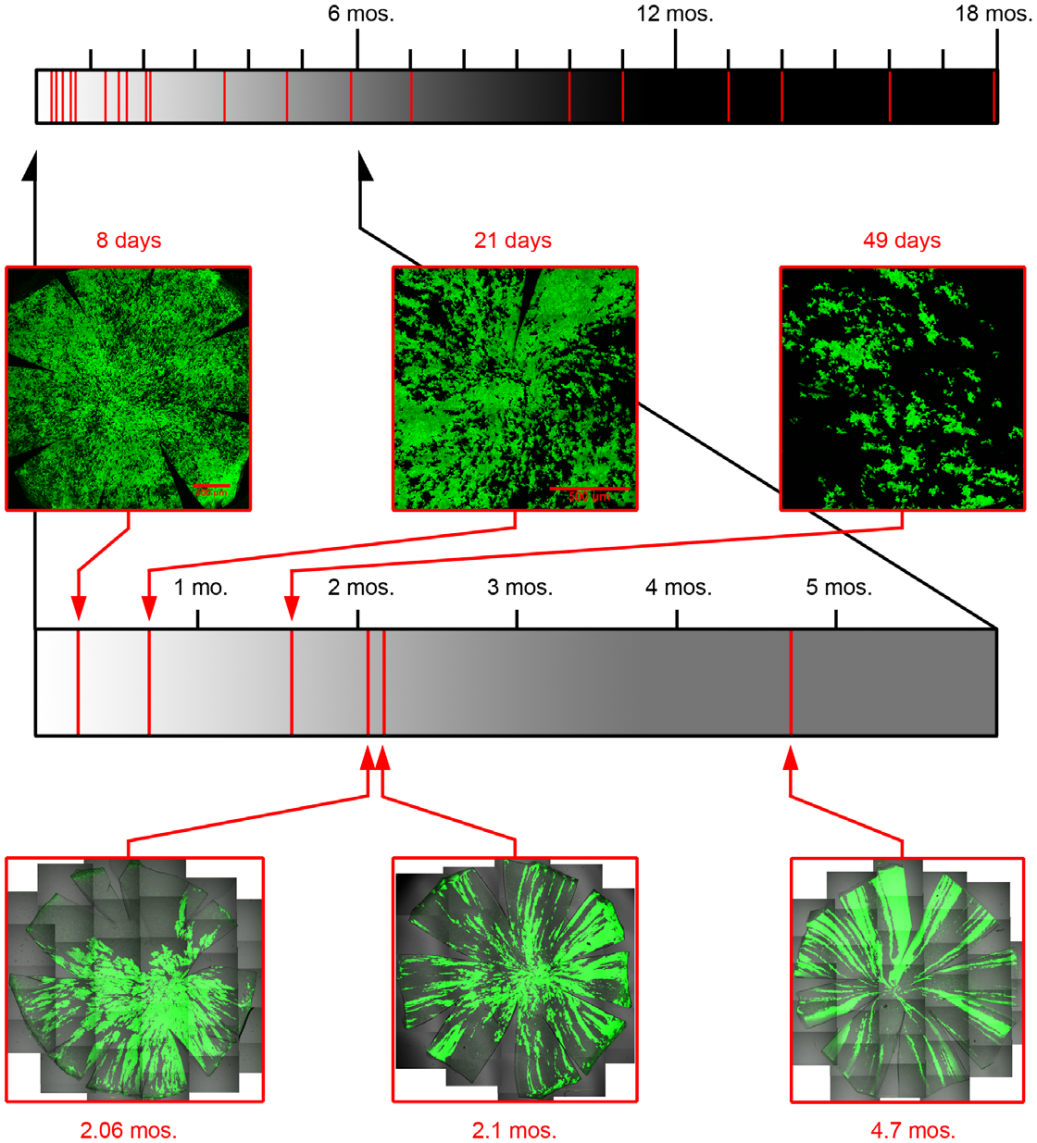
Development of chimeric rat cornea patterning. The transition to stripes and spirals occurs rapidly around 2 months of age. The timeline at the top of the figure displays animal ages where the cornea pattern was examined (vertical red lines) over the range of 8 days-old to 18 months-old. Below is an expanded view of the first 6 months following birth with selected representative images of corneal epitehlium to highlight the rapid transformation of pattern.

**Figure 8:**
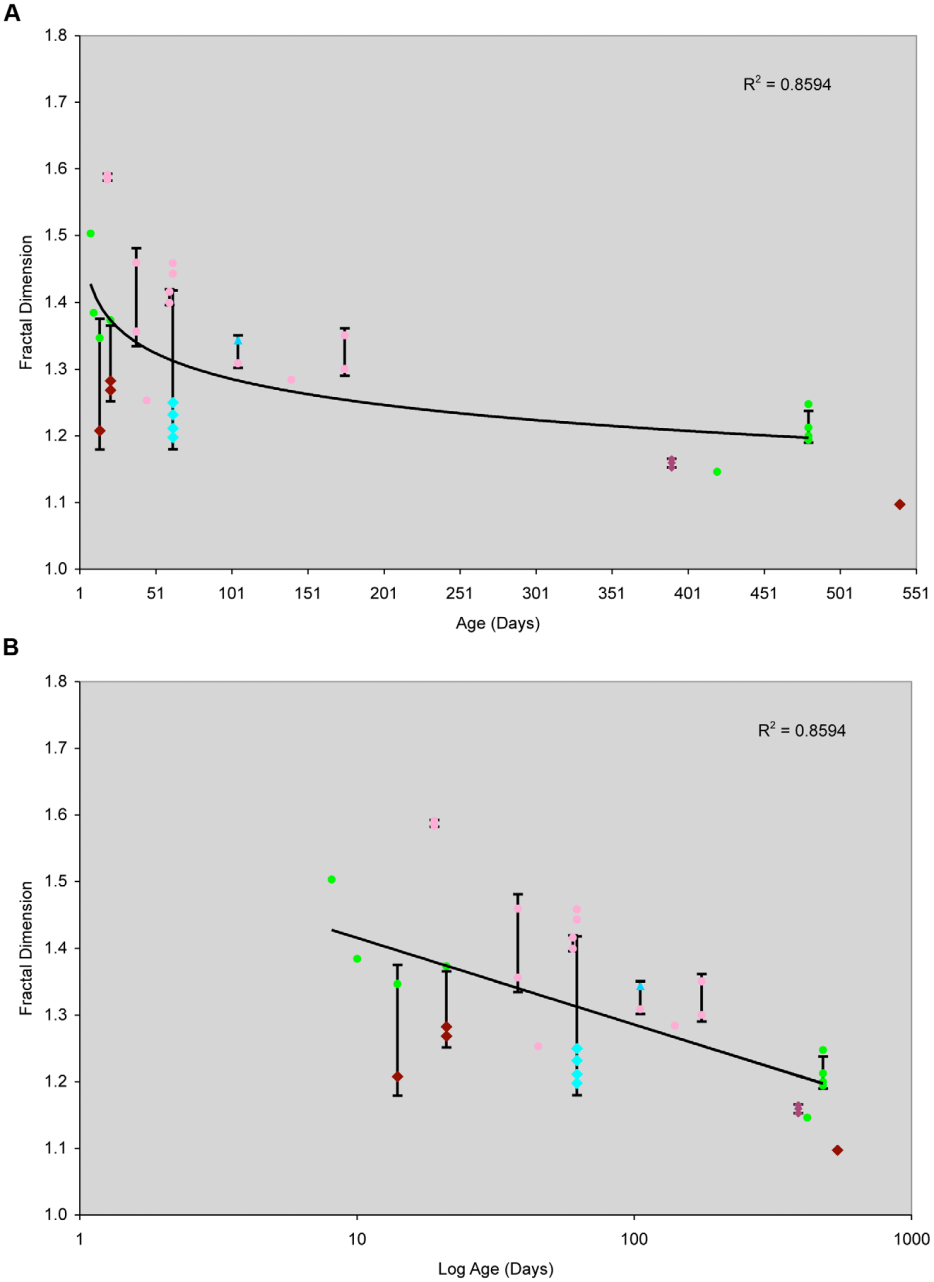
Chimeric rat cornea fractal dimension as a function of animal age. Fractal dimensions were measured for 18 out of 20 chimeric rats spanning ages from 8 days-old to 18 months-old. As the animal ages the cornea pattern shifts from unconstrained geographic to highly constrained spirals. The fractal dimension decreases as the animal ages. The plots displaying this decrease in complexity are shown in a linear scale (A) and semi-log scale for age (B). For cases where more than one image was analyzed for the same age, error bars are shown for ±1 standard deviation from the mean. Because the resolution at which the image was collected can have some influence on the measured fractal dimension, data points are colored based on the original image's resolution.

The spiral pattern is not present from birth, however. At young ages the cornea has a patch pattern similar to that of the liver, “islands in a sea” (Figure 4). The pattern changes to a spiral fairly abruptly beginning at 2 months of age (Figure 7). As the spiral develops the overall shape of the patch becomes constrained and as might be predicted the fractal dimension decreases with age (Figure 8) as the degree of spiral formation increases. The overall average surface fractal dimension of the patches in 9 corneas from animals greater than 2 mo of age is 1.25, while that from animals less than 2 mo of age is 1.39.

### Stereographic Projection

**Figure 9:**
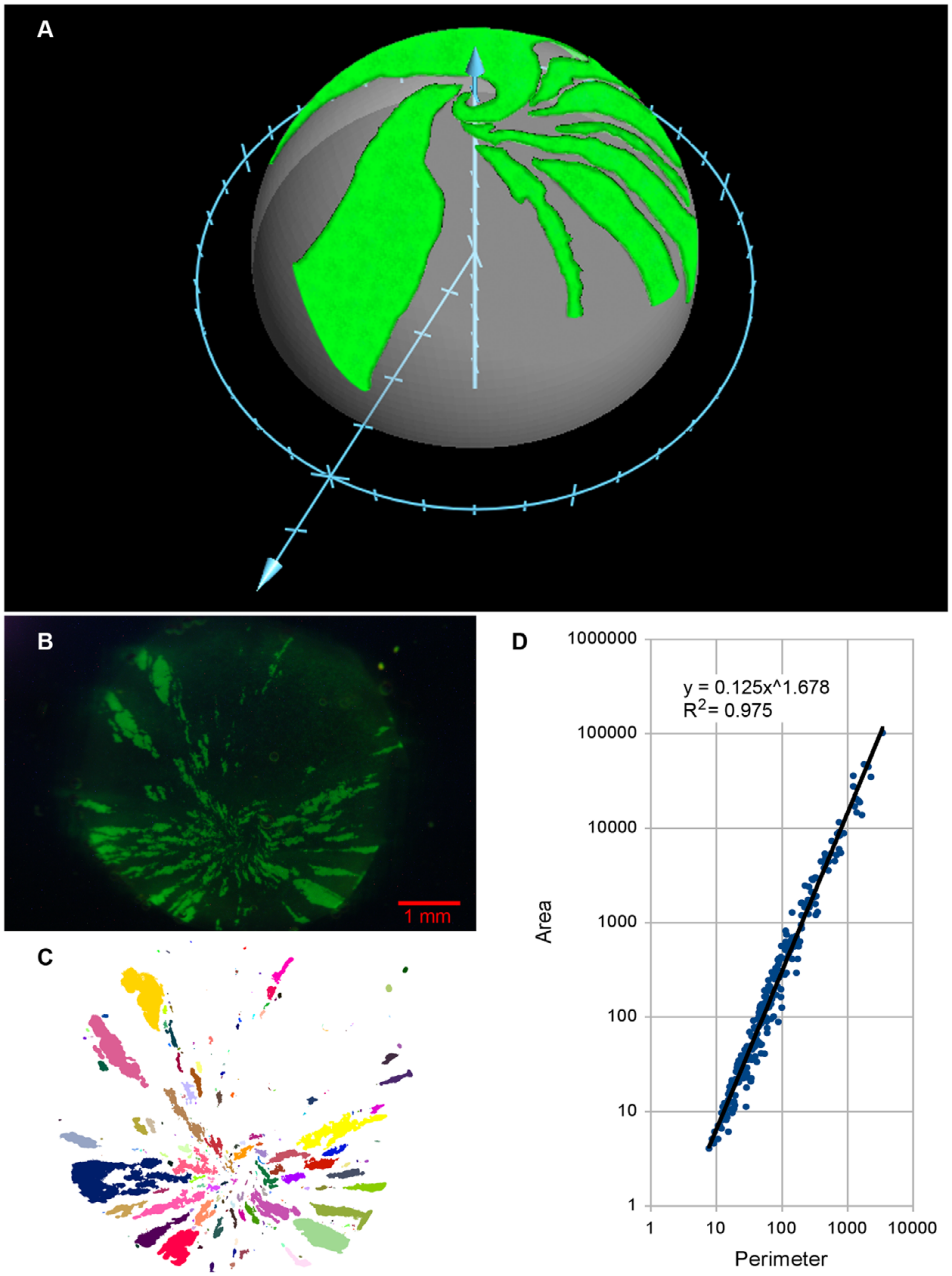
Projection of cornea patch data onto a hemisphere, and unrelaxed chimera cornea patches are fractal. (A) To ensure that no gross deformities in patch geometry occurred as a result of relaxing the corneas prior to imaging them, patch edges from the flattened images were projected back onto a hemisphere. This transformation was done as described in Methods. Edge points were plotted on a hemisphere to show the results in three dimensions. No abnormalities or anomalies were observed. (B) Prior to being relaxed, a 3.5 month-old chimeric rat cornea was photographed (eGFP lineage is green) and the stereomicrograph was converted to a binary image (C) where each patch analyzed was assigned a color. (D) The fractal nature of the corneal patches was determined by examining the relationship of the area and the perimeter of each patch in a log-log plot. This relates the length of the perimeter of each patch measured at the highest resolution with area. When all the objects have ‘coastlines’ sharing the same fractal dimension, the points approach a line in a log-log plot. Furthermore, the slope of the regression line plot of log(area) on log(perimeter) is related to the fractal dimension as D = 2/slope.

The analysis is done with the dome of the cornea flattened to allow the maximum focal plane resolution in confocal microscopy. When projected back onto a hemisphere the potential for shape distortion can be seen to be minimal (Figure 9A), as long as the cut areas are avoided in the analysis. In some cases intact corneas were analyzed, for example Figure 9B,C,D. In this example the data clearly show the patches to be fractal. The intact cornea required photographing with a macro lens due to its size and long exposure times were required. There was an inherent loss of focus, whereas sensitive, high resolution imaging by confocal microscopy was required for accurate determination of the fractal dimension. Thus, direct comparisons of fractal dimensions between these imaging methods are not appropriate.

**Figure 10:**
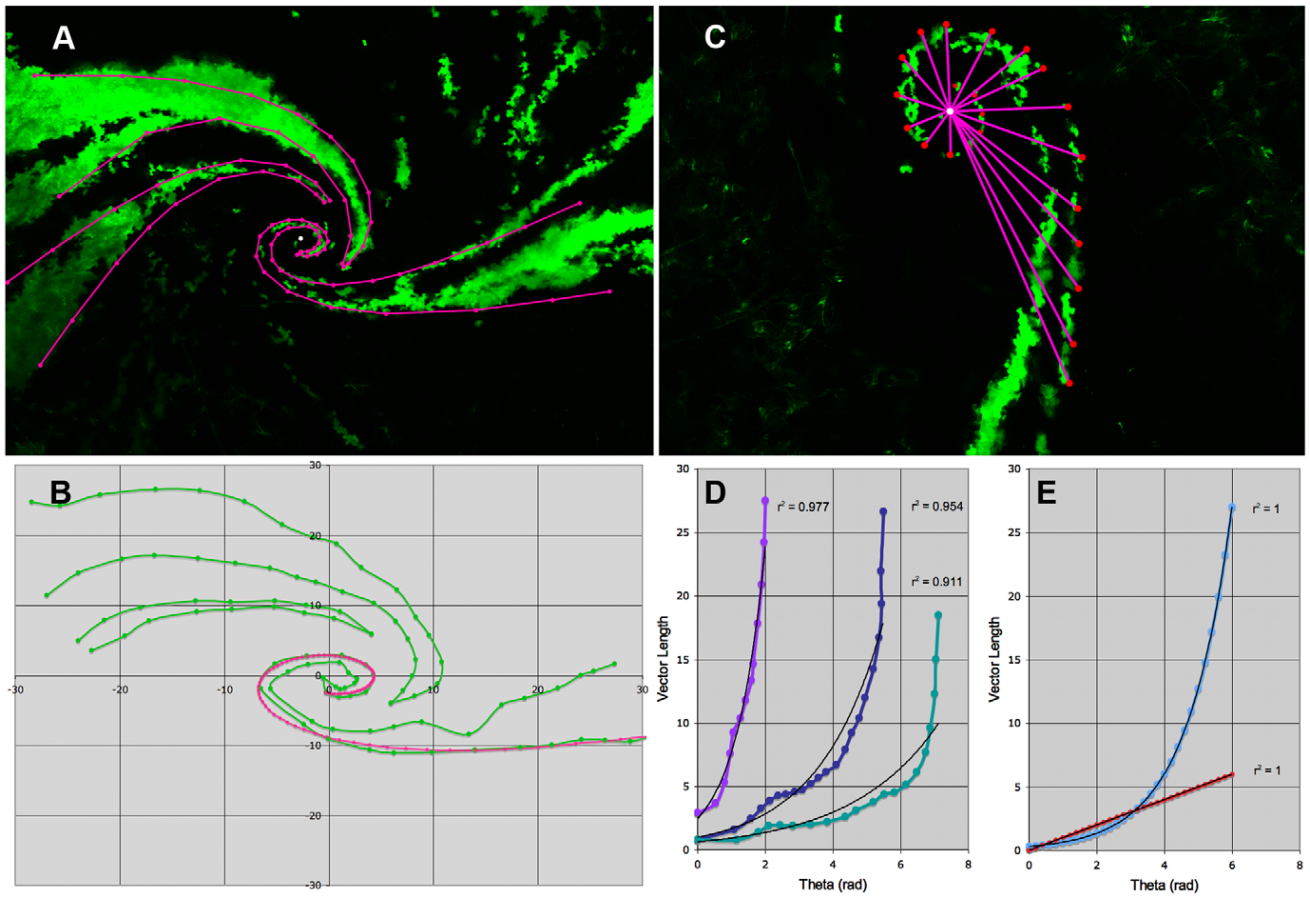
Rat chimera cornea patch edges fit loxodromal spirals and a comparison with logarithmic spirals. (A) A confocal image of a chimeric rat cornea was overlaid with spiral curves, shown in bright purple. Lines connecting the points were drawn in to illustrate the full curve. The white point marks the center of the calculated spirals. The fit of the mathematically generated spiral was performed visually in Excel by adjusting parameters until an acceptable agreement between the spiral and patch edge points collected was seen. (B) The calculated spiral was plotted along with the patch edge points to assess the fit. Parameters of the spiral generated in the figure were as follows: *a = 0.067*, *λ_0_ = −1.2*, *r = 50*, *x_stretch_ = 0.008*, *y_stretch_ = 0.006*, *x_shift_ = 0.5*, and *y_shift_ = −0.5*. This illustration is representative of fits made for 18 patch edges. (C) The distance from the center of a logarithmic spiral to the curve increases exponentially as one proceeds along the curve. A spiral patch is shown. A line was drawn from the center of the spiral to the patch edge at various positions along the patch edge shown in bright purple, and the lengths of these lines (vector lengths) were determined. (D) The vector length was plotted against the angle (theta) defined to be the change in direction from the innermost point along the patch edge in radians. Theta is oriented such that it is increasing regardless of the handedness of the spiral. Data from three representative patches are shown. The data were fit to both an exponential and a linear model, the correlation coefficient (r^2^) for the logarithmic fit is shown. Linear r^2^ values were lower than those of an exponential fit for every case. (E) Correlations were checked for ideal cases of logarithmic and Archimedean spirals generated from equations defining the curves. The logarithmic spiral fit exponentially and the Archimedean fit linearly; both had r^2^ values of 1.00 as expected. Fitting the logarithmic spiral linearly and the Archimedean exponentially yielded much lower values.

The spiraling pattern seen in the cornea exhibits traits similar to those of a loxodrome, which is a curve describing a path to a pole with an invariant non-zero angle to meridians. As a loxodrome approaches the apex, the path becomes a logarithmic spiral. Moreover, the stereographic projection of a loxodrome curve is described by a set of equations to which the data can be fit. In the case of a sphere the stereographic projection of a loxodrome is a logarithmic spiral. Comparing edges of the imaged corneas and projected spirals as seen in Figure 10A,B shows agreement between the predicted behavior of a loxodrome and the patch edges. Eighteen patch edges in 6 corneas from 4 chimeras were analyzed in this way with similar results.

A logarithmic spiral is one in which the distance from the center of the spiral increases exponentially as one moves along the curve. That is, as the angle theta between a line (vector) connecting the center of the spiral and an arbitrary point along the spiral and the vector connecting the center to the curve at points further along the spiral increases, the length of the vector increases exponentially (Figure 10). In an Archimedean spiral by contrast the length of the vector increases linearly. Vectors were drawn to arbitrary points along patch edges; the angle theta and the vector length were calculated for 24 patches, from five corneas in four chimeras (Figure 10C). The vector length was plotted against the angle theta and the data were fit to both an exponential and linear function. Three representative plots are shown in Figure 10D. The correlation coefficients for an exponential fit for the 24 patches varied from 0.77 to 0.99, and in all cases were greater than the correlation coefficient for a linear fit. The same procedure was applied to an ideal logarithmic spiral and an ideal Archimedean spiral both generated from equations defining the curves. The correlation coefficients of the exponential fit of the vector lengths of a logarithmic spiral and the linear fit of the vector lengths of an Archimedean spiral were in both cases 1.00 (as expected). Thus, Archimedean and logarithmic spirals can be discriminated by the relation between vector length and angle.

## Discussion

Here we have shown that fluorescent markers can be used to provide high-resolution images of patches in a variety of tissues. These patterns are conserved and regulated and can be reconstructed in rendered three-dimensional images suitable for analysis. The patterns observed are a reflection of the forces that could give rise to them; e.g., cell division patterns, cell movement patterns and cell death. However, computer simulation previously has shown that the complicated mosaic patterns observed do not strictly require cell movement, but rather can reflect self organizational attributes of repetitively applied cell division rules or algorithms, at least in the liver and adrenal cortex. Patches themselves reflect a dynamic state of oscillation in size as patches grow clonally and then fragment followed by renewed patch membership as cells are pushed back into previous patches. The equilibrium state therefore represents the output of a dynamical system. A prediction of this model is that the patch geometry will display fractal characteristics. In particular, the intriguing pattern of spiral patches observed first in mice is shown here to be conserved in the rat using a transgenic marker unrelated to the cornea. This supports the idea that the pattern observed is due to cell assortment of the two parental lineages that formed the chimeras and not developmentally regulated expression of the marker [1], [25].

Although mouse chimeric and mosaic transgenic studies have been fruitful for examining some aspects of corneal epithelial spiraling, the underlying mechanism remains unclear. Fixed examinations of mosaic corneas at different times demonstrate a steady state of randomly dispersed distribution transitions to a whorl pattern over several weeks [11], [26]. Based on discrete measurements, West and colleagues proposed that spirals result from “coherent” clonal patches (descendants that remain stuck together) emanating from the limbus, which migrate centripetally [27]. While examinations of mature corneal epithelial cells *in vivo* support aspects of this explanation [28], the less common forms (branching patches, patches that don't make it to the apex and arc shaped patches) are more difficult to explain in this way. Another problem is that measured migration rates are too slow to explain the speed of emergence of the spiral pattern from the initial “islands in a sea” pattern [27]–[31]. That is with a measured migration rate of 17 µm/day [31] and a corneal diameter of 2.3–2.6 mm [32] migration would take about 2–2.5 mo from the limbus to the apex, while the pattern changes from patchy to striped in less than 3 weeks [30]. In the rat the migration at that speed would take closer to 6 mo, while the transition occurs in less than 1 mo. Moreover, it is generally held that population pressure alone cannot explain the cell migration [33]. Additionally, it is clear that cell divisions generating corneal epithelium are not restricted to the limbus [34]. This is an area of some controversy [35] but in individuals with limbal stem cell deficiency normal cornea with polygonal superficial cells, well-defined wing cells, and basal cells develop and are maintained [36].

In transgenic animals the marker gene can be silenced stochastically by as yet poorly understood mechanisms. This process is likely due to position effects depending on integration sites. Regardless of the manner by which silencing occurs it can result in the formation of mosaic pattern on its own [16]. However, such silencing is not a uniform property of transgenic animals or the same in all tissues and in this study we have selected a transgenic strain with high levels of uniform marker expression. Control corneas only show gene silencing in less than 1% of the areas examined. In chimeras made from this strain the effect of gene silencing on the observed patterns in the cornea is negligible. We have shown previously that the patterns observed in adrenal cortex in aggregation rat chimeras and transgenic rat and mouse that are mosaic because of gene silencing are qualitatively and quantitatively very similar [10].

Imaging the cornea required sufficient sensitivity to visualize the fluorescent signal from the eGFP labeled cells along with a high level of resolution to discriminate cellular details of the patch edges. In addition a large field of view was needed so that the whole area of the cornea could be imaged. Confocal imaging was ideal with both high sensitivity and high resolution. In conjunction with a computer controlled motorized stage, many fields of view could be imaged and tiled to cover the entire area of the cornea. However, confocal microscopy only images a plane of focus in the specimen and therefore the corneas needed to be flattened. This was achieved by cutting small, radial incisions along the perimeter of the cornea to allow it to lay flat with little deformation. The epithelium, stroma, and endothelium now lay in separate planes of focus for the entire cornea, making analysis of fluorescent signal from the different layers a matter of changing the focus of the microscope. In the intact cornea discriminating fluorescence from the distinct layers of the cornea becomes increasingly difficult toward the periphery. A number of other issues of imaging a large round surface microscopically, including an increasingly steep angle of incidence for laser illumination are problematic. Thus precise imaging of the cornea leads to distortion either from cutting the cornea or from optical distortion in imaging the intact hemisphere. We chose to flatten the tissue because of the benefit of allowing the best resolution for the patch edges from the center of the cornea to the limbus necessary to measure fractal dimension. By cutting the cornea at the periphery, the displacement of patches was least at the center of the tissue and of course the cut edges can be eliminated from analysis. The center is also where a loxodrome most closely approximates a logarithmic spiral.

Fractal objects are those in which great detail is nested within detail. As one examines the object at closer distances or at higher magnification more detail than is predicted from more distant observation is revealed. It is not merely a matter of resolution but rather is an inherent property embedded in the object. As one looks at one part of a fractal it closely resembles all other parts. In mathematical terms fractals are said to be self-similar and to scale. If one measures the perimeter, area or volume of a fractal at different magnifications these measurements will change more than would be expected from the difference in magnification [18], [37]. Euclidian objects like circles or squares are not fractal. Examination of these objects at different magnifications show that measurements of perimeter, area or volume change as predicted from the change in magnification. Fractal objects are widespread in nature; examples include rivers and shore lines. Consider the length of a river or shoreline. It is not an absolute, It depends on the distance from which the measurement is made. Observing from an airplane gives a much shorter length than by walking along in and out among rocks and bays. Previously we established that mosaic patches in chimeras are fractal [5]. The implication of this observation is that an iterative and recursive process guided by cell division might be enough to explain their development. This was further demonstrated for liver and adrenal patterns by means of computer simulations [23]. Fractals can be described with a statistic called the fractal dimension, which is a measure of geometric complexity in the object [4], [38]. In this study we have used the fractal dimension to quantitatively establish the dynamics of pattern transition in the corneal epithelial cell assortment. Since the epithelium is at first patchy with a high fractal dimension one might conclude the pattern arose from the effects of cell division as in the liver where a dynamic process of cell division, daughter cell placement, and bumping of existing cells results in patches that grow until incursion from neighboring patches of the other lineage fragments them and then achieves an equilibrium as bumping pushes patches of like type together again [4]. However, as the spiral develops the fractal dimension dramatically falls suggesting that a different process is responsible. In the adrenal gland we have previously shown that while the fetal patches are island-like, as in the liver, the transition to the adult pattern of radial cords does not result in a lower fractal dimension [10].

Fractal analysis is the approach of choice for the characterization of patch boundaries for two reasons. Firstly, it provides a quantitative parameter of the extent of cell population mixing which is robust to relative proportions of the cell types. This was shown in chimeric tissues (for example figure 3e in reference [4]) as well as in computer simulations [23]. Secondly, since the mosaic patches are dynamic structures that reveal the net result of iterations of cell death and their replacement at the expense of other cells, the patches end up becoming geometrically complex and cannot be systematically characterized by (for example) a single boundary length or patch area alone as these parameters depend on the scale of observation (and the latter also on the original proportion of cell lineages in the tissue, e.g. see figure 2c in reference [4]). It is this dependence on resolution that requires the multi-scale characterization that fractal geometry provides.

Spirals are a class of curves whose radius continuously increases. D'Arcy Thompson in On Growth and Form noted that of the many types of spirals the two most important are Archimedean and logarithmic [39]. In an Archimedean spiral the distance between the center of the spiral and the curve increases linearly as one proceeds along the curve while in a logarithmic spiral that distance increases geometrically or exponentially. The latter result was obtained here for corneal patches.

Natural spirals often take on the geometry of logarithmic spirals that are self-similar where each curve increases in scale but not shape. By definition the spirals we see in the rat corneal epithelium are not logarithmic as many have an inflection where the direction of rotation changes. However, the curve describing the patch edge approximates a logarithmic spiral, with simple transformations, and the radius of the curve increases exponentially for most of the patch length between the center and the inflection consistent with a loxodrome. A loxodrome is the most efficient path to the apex of a hemisphere insofar as the direction of the cell movement does not change along the path to the apex. While the shortest path to the apex is straight along a meridian, the loxodrome may emerge from directional uncertainty, an inability for the assorting cells to change direction or a perturbation of direction at some point in the process. One might speculate that since both loxodromes and straight stripes are seen in the rat cornea that some global force is responsible for the epithelial cell assortment. The path of a loxodrome is an explanation of the spiral assortment that does not require rotation of the tissue during development. Further if some perturbation of the direction of cellular assortment occurs again without the ability of the cell to change the direction of movement, then the pathway traced to the apex is a loxodrome, while if unperturbed the shortest path to the apex would be straight along a meridian. If such perturbation were stochastic then there should be random distribution of patterns and some individuals should have both clockwise and counter-clockwise spirals as was indeed observed. The idea of a global force driving the cell assortment is consistent with the observation that subepithelial nerves tend to form pinwheel distribution patterns on about the same time scale as epithelial cell assortment [26], [40].

Even though spirals are present in many forms in nature and have attracted the attention of many investigators, general principals of their formation are lacking. Energy minimization attributes of spiral organization may be significant in developmental settings like the cornea where stress and strain have consequences for function. The cornea may prove to be useful as a model of how and why these fascinating structures develop.

## Supporting Information

Figure S1 **Three-dimensional reconstruction of chimeric liver animation.** Liver from a chimeric rat was sectioned at 35 µm and imaged with confocal microscopy. One plane of focus was used to represent each 35 µm section. These sections were then stacked and aligned to render a three dimensional model illustrating the complex interconnected nature of the patches. Shown here is an animation of the model being rotated. Total area shown is approximately 4 mm by 4 mm. eGFP lineage is green.
(MP4)

Figure S2 **Detailed three-dimensional reconstruction of chimeric liver animation.** A three dimensional rendering of chimeric rat liver was produced in the same manner as described in Figure S1 with the exception that sections were imaged at higher magnification to illustrate the detail of fluorescent patches. Shown here is an animation of the model being rotated. Total area shown is 0.35 mm by 0.35 mm. eGFP lineage is green.
(MP4)

Figure S3 **Three-dimensional reconstruction of chimeric rat adrenal gland animation.** Sections of a chimeric rat adrenal gland were cut at 35 µm and imaged at one focal plane with confocal microscopy. These images were then stacked and aligned to construct a three-dimensional rendering. This highlights the radial cord-like structure of the fluorescent patches in the adrenal cortex, which are reminiscent of pencils in a cup. Shown here is an animation of the rendered image being rotated. Total area shown is approximately 4 mm by 4 mm. eGFP lineage is green.
(MP4)
